# Anti-NMDAR encephalitis induced in mice by active immunization with a peptide from the amino-terminal domain of the GluN1 subunit

**DOI:** 10.1186/s12974-021-02107-0

**Published:** 2021-02-21

**Authors:** Yuewen Ding, Zheye Zhou, Jinyu Chen, Yu Peng, Haitao Wang, Wei Qiu, Wei Xie, Jun Zhang, Honghao Wang

**Affiliations:** 1 Department of Neurology, Nanfang Hospital, Southern Medical University, 1838 North Guangzhou Avenue, Guangzhou, Guangdong 510515 People’s Republic of China; 2 School of Traditional Chinese Medicine, Southern Medical University, 1838 North Guangzhou Avenue, Guangzhou, 510515 Guangdong People’s Republic of China; 3 School of Biomedical Engineering, Liuzhou Traditional Chinese Medicine Hospital, Guangzhou, Guangdong People’s Republic of China; 4 School of Pharmaceutical Sciences, Southern Medical University, Guangzhou, Guangdong People’s Republic of China; 5 Department of Neurology, Third Affiliated Hospital of Sun Yat-Sen University, Guangzhou, Guangdong People’s Republic of China; 6 Department of Internal Medicine, Division of Nephrology, University of California at Davis, Houston, TX USA

**Keywords:** Anti-NMDA receptor encephalitis, Amino-terminal domain, GluN1, Active immunization, Cerebrospinal fluid

## Abstract

**Background:**

Anti-N-methyl-D-aspartate receptor (NMDAR) encephalitis is a recently discovered autoimmune syndrome associated with psychosis, dyskinesia, and seizures. However, the underlying mechanisms of this disease remain unclear, in part because of a lack of suitable animal models.

**Methods:**

This study describes a novel female C57BL/6 mouse model of anti-NMDAR encephalitis that was induced by active immunization against NMDARs using an amino terminal domain (ATD) peptide from the GluN1 subunit (GluN1_356–385_).

**Results:**

Twelve weeks after immunization, the immunized mice showed significant memory loss. Furthermore, antibodies from the cerebrospinal fluid of immunized mice decreased the surface NMDAR cluster density in hippocampal neurons which was similar to the effect induced by the anti-NMDAR encephalitis patients’ antibodies. Immunization also impaired long-term potentiation at Schaffer collateral–CA1 synapses and reduced NMDAR-induced calcium influx.

**Conclusion:**

We established a novel anti-NMDAR encephalitis model using active immunization with peptide GluN1_356–385_ targeting the ATD of GluN1. This novel model may allow further research into the pathogenesis of anti-NMDAR encephalitis and aid in the development of new therapies for this disease.

**Supplementary Information:**

The online version contains supplementary material available at 10.1186/s12974-021-02107-0.

## Background

Anti-N-methyl-D-aspartate receptor (NMDAR) encephalitis is an autoimmune disorder of the central nervous system (CNS) that predominantly affects young females. The typical clinical manifestations include progressive development of neurologic and psychiatric symptoms, including abnormal movements, seizures, impaired memory, and behavior disorders [1]. Nearly 80% of patients show CSF laboratory abnormalities, presenting as mild lymphocytic pleocytosis and normal or moderately increased protein levels and 60 % of patients have specific CSF oligoclonal bands [2, 3].

It is believed that the ectopic expression of NMDARs contributes to triggering the immune response [4]. Antigens are taken up by antigen-presenting cells that travel to regional lymph nodes, and plasma cells produce antibodies (ABs) that later react with NMDARs in the brain, impairing blood-brain barrier permeability [5]. NMDARs mediate glutamatergic synaptic transmission and play a prominent role in synaptic plasticity. Previous studies have suggested that the pathogenicity of autoimmune ABs is a key mechanism of anti-NMDAR encephalitis [1, 6]. Human CSF-derived NMDAR ABs were shown to downregulate NMDAR levels in both in vitro and in vivo studies [7, 8]. In addition, reduced NMDAR expression can result in increased extracellular glutamate and thus affect the pons/medullary respiratory center [7].

Passive immunization in mice, using intrathecal infusion of CSF from affected humans, leads to behaviors of depression-like behaviors, anhedonia, and memory deficits. Although these studies provide compelling evidence of the pathogenicity of antibodies, it is unable to show the process of autoantibody production in vivo [9, 10]. Thus, an animal model of active immunity is needed that more closely mimics disease progression.

Previous investigations have highlighted the extracellular amino-terminal domain (ATD) of the GluN1 subunit, especially the N368/G369 region, as essential for immunoreactivity [11, 12]. Therefore, ATD peptides were used to immunize mice. Twelve weeks later, the mice demonstrated behavioral changes and AB infiltration, which was most prominent in the hippocampus. The presence of GluN1 ABs and their effect on NMDARs were also confirmed.

## Methods

### Study design and mice immunization

The study aimed to investigate the effects of active immunization with NMDAR peptides in normal adult mice. C57BL/6 mice (10 weeks old, female) were immunized with different GluN1 extracellular peptides emulsified in an equal volume of Complete Freund’s Adjuvant (CFA) supplemented with *Mycobacterium tuberculosis* H37Ra (4 mg/mL) at a final peptide concentration of 1 mg/mL. Mice were immunized subcutaneously on the back with 200 μg of peptide in the emulsion mixture and received two booster injections with peptide emulsion at 4 and 8 weeks after the first immunization. Mice in control group received emulsion mixture of CFA and equal volume of phosphate-buffered saline (PBS). All mice were intraperitoneally injected with 200 ng of pertussis toxin (List Biological Laboratories) on the day of the last immunization and 48 h later.

To detect antibody titer, cerebrospinal fluid and serum were obtained from three mice and tested every other week. Behavioral tests and histological staining were performed 12 weeks after the first immunization.

### Patient sample collection

We collected CSF from patients with high titers of anti-GluN1 ABs (> 1:300) during routine clinical examinations. All patients fulfilled the clinical diagnostic criteria for anti-NMDAR encephalitis, revised in 2016 [13]. The study protocol was approved by the ethics committee of Nanfang Hospital, Southern Medical University, and written informed consent was obtained from each participant or their guardian.

### Antibodies purification

CSF ABs from patients or immunized mice were purified using protein G Sepharose columns and were then used to treat neurons or brain slices. Considering the low amount of CSF obtained from an individual mouse, we pooled the CSF of 40 mice for further procedures. For the purification process, 2 mL of sample diluted with PBS was incubated in a chromatography spin column (Thermo Scientific) of protein G Sepharose beads for 30 min. After three washes with PBS, the samples were eluted with elution buffer, dialyzed against PBS, concentrated in stock solutions of 4 mg/mL, and stored at – 80 °C until use.

### Preparation and staining of GluN1-expressing HEK cells

Human embryonic kidney 293 (HEK293) cells were transiently transfected with NMDAR subunit genes (NR1/NR2A; DsRed2-labeled) as previously described [14]. Twenty-four hours later, cells were fixed on coverslips with acetone and incubated overnight at 4 °C with the purified ABs from patients or CSF from immunized mice (starting at a 1:1 ratio) in PBS containing 5 % bovine serum albumin (BSA). After washing with PBS, the cells were labeled with fluorescein isothiocyanate (FITC)-conjugated anti-human IgG (ab7149, Abcam) or FITC-conjugated anti-mouse IgG (ab6785, Abcam) and observed under a fluorescence microscope (BX51, Olympus, Japan).

### Site-directed mutagenesis

A point mutation was generated using the Stratagene QuikChange Mutagenesis kit (210518, Agilent) according to the manufacturer’s instructions. The following primers were designed for the N368Q point mutation: Forward: 5′-gggatgacatgggtaccttggtagatg cccacttgca-3′; Reverse: 5′-tgcaagtgggcatctaccaaggtacccatgtcatccc-3′.

### Primary neuronal cultures

Hippocampal neurons from embryonic day 18 rat brains were prepared and maintained as previously described [15]. The hippocampi were dissociated with papain at 37 °C for 30 min, and then separated with a fire-polished Pasteur pipette. After centrifugation at 300×*g*, the cells were resuspended in Neurobasal medium. Cells were counted and plated onto poly-D-lysine-treated 24-well plates. After 6 h, the supernatant was removed and replaced with 500 μL of fresh culture medium. Cells were then cultured for 14 days for subsequent experiments.

### Immunocytochemistry

Immunocytochemistry was performed to detect autoantibody 12 weeks after immunization with GluN1_356–385_ peptide or a control peptide GluN1_369–386_ which was unable to induce specific ABs. The mouse brains were cut into 15 mm slices, permeabilized with 0.5% Triton X-100, preincubated with 10% normal goat serum, and incubated with fluorescent secondary ABs (1:200, anti-Mouse Alexa Fluor 488, A-11029) to detect autoantibodies. The slices were imaged using a confocal microscope (LSM 880, Carl Zeiss, Germany). To stain surface NMDARs, primary hippocampal neurons were incubated with ABs (5 μg/mL) derived from the CSF of patients or immunized mice. After 18 h of incubation, the AB-bound surface receptors were incubated with fluorescently conjugated secondary ABs (1:200, anti-Mouse Alexa Fluor 488, A-11029; 1:200, anti-Human Alexa Fluor 488, A-11013; Invitrogen) at room temperature for 1 h. Cells were then fixed with methanol at – 20 °C for 5 min, permeabilized with (0.5%) Triton X-100 for 10 min, blocked in 10% normal goat serum for 30 min and incubated with anti-PSD95 primary AB (1:200, Synaptic Systems, 124-003) at 4 °C overnight to label postsynaptic densities, and visualized after staining with Alexa Fluor 647 (1:200, Invitrogen, A-21244) for 1 h at room temperature.

### Electrophysiological recording

Brain slice preparation and electrophysiological recording were performed as previously described [16]. Mouse hippocampal slices (300 μm) were prepared using a vibratome (VT1000S, Leica, Germany). The slices were kept at 30 °C for at least 60 min before experiments in artificial CSF (ACSF; NaCl 124 mM, KCl 2.5 mM, MgSO_4_ 2.0 mM, NaH_2_PO_4_ 1.25 mM, NaHCO_3_ 26 mM, CaCl_2_ 2 mM, and glucose 10 mM; pH 7.3), bubbled with a mixture of 95% O_2_ and 5% CO_2_. Field excitatory postsynaptic potentials (fEPSP) were evoked in the CA1 stratum radiatum by stimulating Schaffer collaterals with a two-concentric bipolar stimulating electrode, and were recorded with ACSF-filled glass pipettes. LTP was induced by applying theta burst stimulation. Purified ABs were diluted in ACSF (100 μg/mL) and applied by switching the perfusion from the control ACSF to the AB-containing ACSF. For each recording, the baseline synaptic transmission was monitored for 10 min before AB perfusion, and AB-containing ACSF was continuously washed out with ACSF after theta burst stimulation until the end of the experiment. For immunized mice, fEPSP were evoked in brain slices in the same way and recorded for 20 min as the baseline prior to the induction of LTP. The acquired data were analyzed with pClamp 10 software (Axon Instruments, USA).

### Calcium imaging

Hippocampal neurons were incubated with 20 μg/mL of patient or mouse ABs at 37 °C for 18 h. To detect calcium flux, the cells were loaded with Fura-2 (1 μM, Invitrogen, F1221) and incubated at 37 °C for 15–30 min, followed by a 15–30-min incubation at room temperature. After washing in Tyrode’s solution, the cells were transferred into wells containing NBQX (10 μM, Tocris, 0373). NMDA (10 μM, Sigma, M3262) was used to stimulate NMDAR-mediated calcium influx. Imaging was performed using an inverted fluorescence microscope (Eclipse TE2000-U, Nikon, Japan) with a charge-coupled device camera. Image series of images were acquired at 800 ms intervals for 40 s at an excitation wavelength of 470 nm. All data were obtained from five independent samples. The fluorescence intensity at each timepoint was measured using ImageJ software (NIH).

### Behavioral assessments

Twelve weeks after immunization, mice were tested using a series of behavioral experiments. Behavioral testing was conducted at uniform times, from 09:00 to 12:00, by researchers blinded to the group allocations. Behavioral parameters were recorded using a video tracking system (Smart 3.0, Panlab, Spain). Details of behavioral tests are described in the Supplementary Methods.

### Western blot analyses

Minute Plasma Membrane Protein Isolation Kit (SM-005, Invent Biotechnologies) was used to extract membrane and total cell proteins from the hippocampus and primary neurons according to the manufacturer's protocol. Forty micrograms of the membrane protein or total cell protein sample was mixed with sodium dodecyl sulfate (SDS) sample buffer and boiled at 98 °C for 10 min. Samples were separated by electrophoresis on 10% SDS-polyacrylamide gels and transferred to polyvinylidene fluoride membranes. After blocking with 5% BSA, the membranes were incubated with anti-GluN1 ABs (1:500, Synaptic Systems, 114-011) and anti-β-actin ABs (1:2000, Proteintech, 20536-1) overnight at 4 °C, washed three times and then incubated with horseradish peroxidase-conjugated anti-mouse or anti-rabbit secondary ABs (1:2000; Proteintech) for 1 h at room temperature. The immunocomplexes were detected using enhanced chemiluminescence (Thermo Scientific).

### Statistical analyses

Data are presented as mean ± SEM. Independent samples *t* tests or Mann–Whitney *U* tests were used as appropriate for each experiment. Kruskal-Wallis test was used to analyze nonparametric data. All analyses were performed using SPSS V24.0 (IBM, USA) and the histograms were plotted using GraphPad Prism 6.0 (GraphPad Software, USA) or ImageJ. A value of *P* < 0.05 was considered statistically significant.

## Results

### Detection of antigen specificity of peptide-induced autoantibodies

**Fig. 1:**
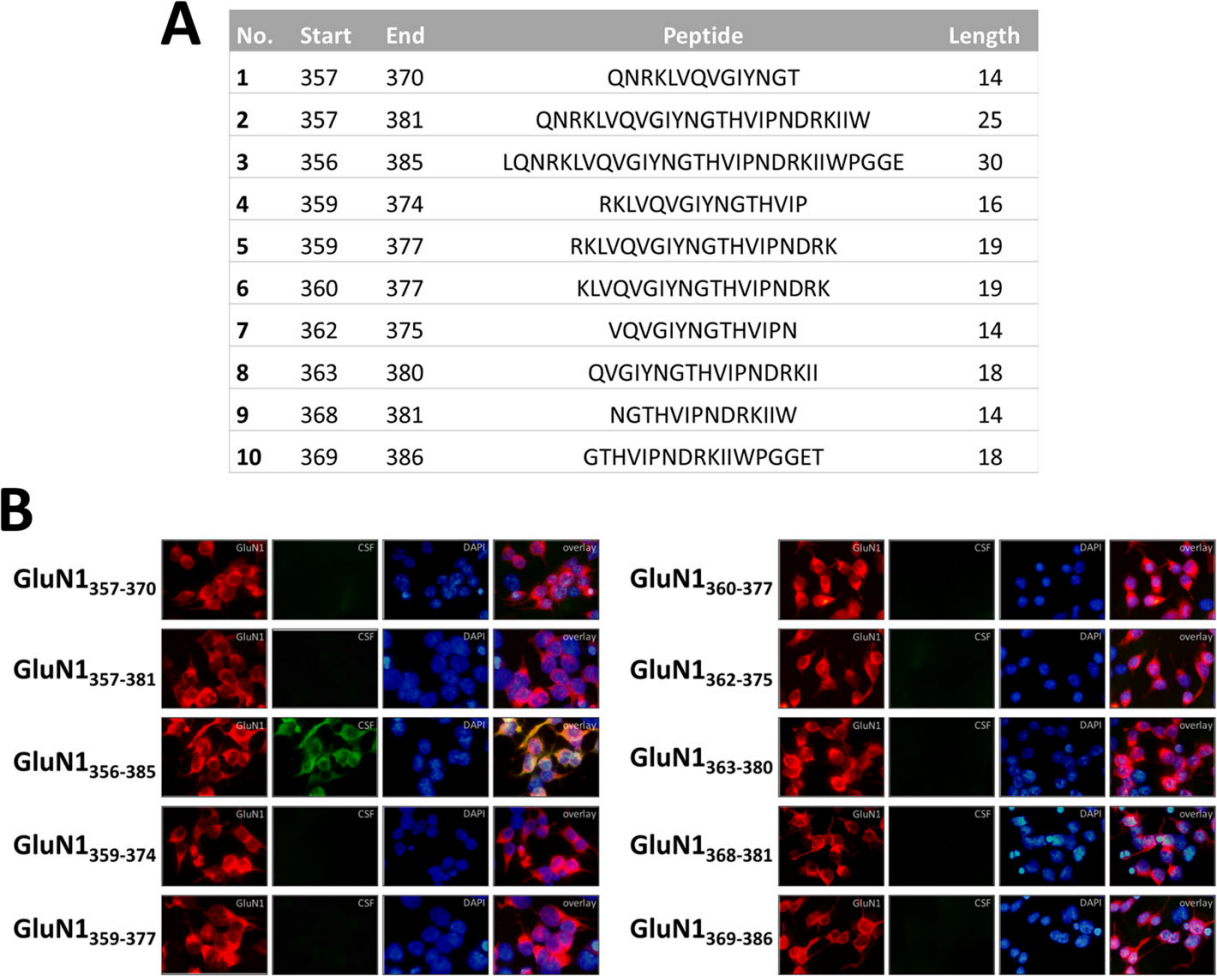
Characterization of ABs from the CSF of mice immunized with peptides of the GluN1 subunit. **a** The peptide sequences used in the immunization experiment. **b** Immunofluorescence detection of GluN1 ATD peptide induced ABs against GluN1 protein using HEK293 cells

To induce an active immune mouse model of anti-NMDAR encephalitis, mice were immunized by subcutaneous injection with 200 μg of peptide emulsified in Complete Freund’s Adjuvant (Fig. 1a). CSF from peptide-immunized mice was collected 14 days after immunization. The CSF ABs were purified and detected in HEK293 cells transfected with GluN1 subunits (DsRed2-labeled). We found that only the GluN1_356–385_ peptide could induce CSF autoantibodies that were specific for GluN1 (Fig. 1b).

### Binding properties of GluN1_356–385_ peptide-induced ABs and their effects on NMDARs in vitro

**Fig. 2:**
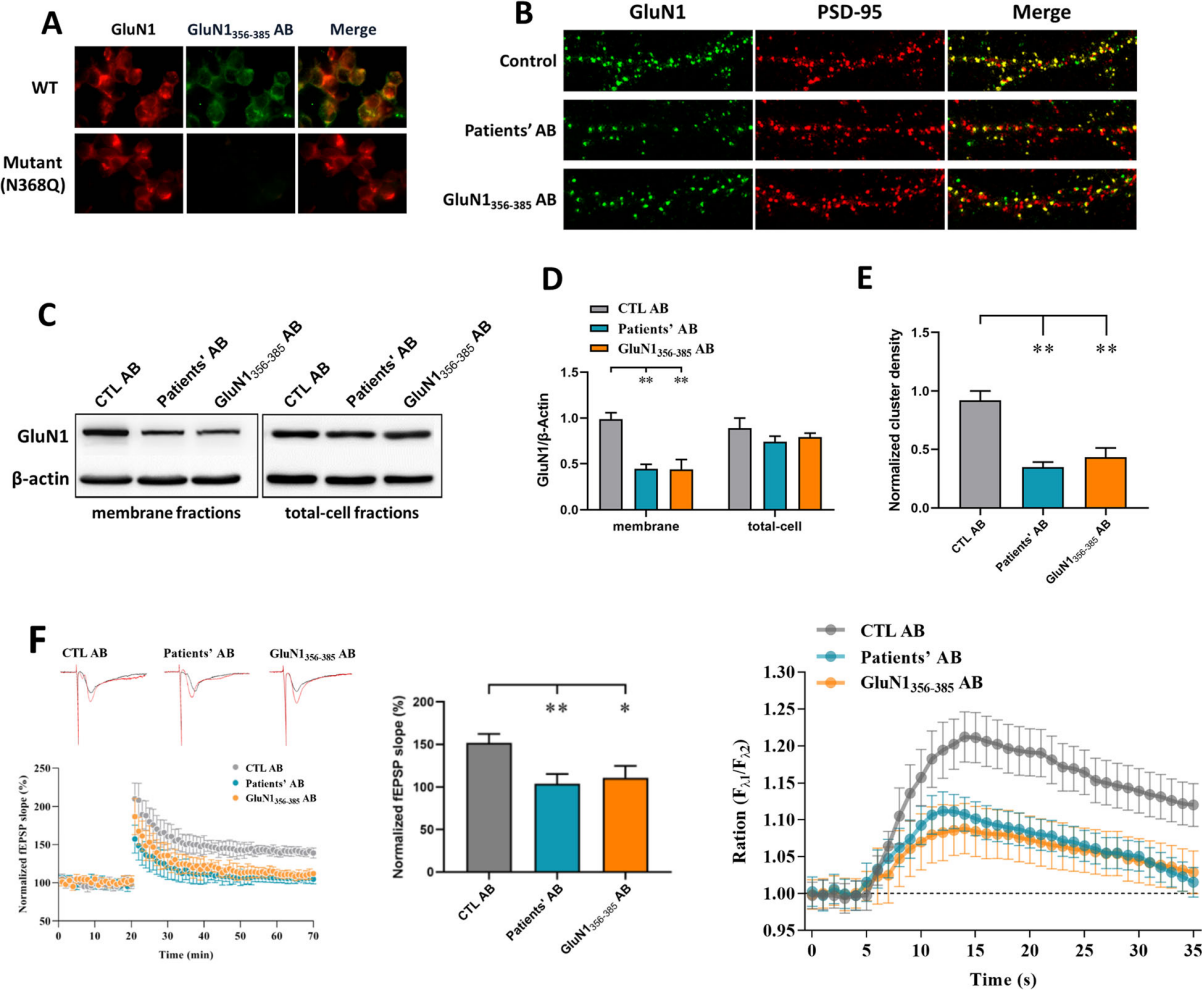
Epitope analysis of GluN1_356-385_ ABs and their effects in vitro. **a** ABs were applied to HEK293 cells transfected with wild-type GluN1 or a construct with mutated amino acid 368 (N368Q). **b**, **e** Hippocampal neurons were immunostained for surface GluN1and PSD95 after treatment for 24 h with patients’, GluN1 ABs, or control ABs and imaged with confocal microscopy. **c**, **d** Western blot analysis of GluN1 on membrane fraction and total cells. **f** Electrophysiological effects of patients’ AB and GluN1_356–385_ AB on tetanus-induced LTP on brain slices (**f**, left) (*n* = 5). Example traces of fEPSPs recorded before (black) and after (red) TBS (**f**, left, top). Normalized slope of field excitatory postsynaptic potentials in hippocampal slices after ABs treatment (**f**, right). **g** Effects of patients’ AB and GluN1_356–385_ AB on NMDAR-mediated calcium influx (*n* = 5). *P < 0.05; **P < 0.01

Binding of ABs to GluN1 from human patients can be prevented by a single amino acid mutation in the ATD of GluN1 (N368Q) [11]. We therefore generated a GluN1 subunit construct with a mutation at amino acid 368 (N368Q) and tested the GluN1-specific clones for their reactivity in transfected HEK293 cells transfected with the mutant construct. The results showed that binding to the mutant was eliminated for the GluN1_356–385_ ABs (Fig. 2a).

The binding of the GluN1_356–385_ ABs to GluN1 led to the question of whether GluN1_356–385_ ABs mediate the internalization of NMDARs in neurons. We therefore incubated primary murine hippocampal neurons with purified human or mouse CSF ABs. AB binding resulted in a marked downregulation of NMDAR-positive synaptic clusters (Fig. 2b, e). Western blots of GluN1 also revealed its significantly reduced amount in the membrane fractions of GluN1_356–385_ AB-treated neurons, indicating a profound loss of synaptic NMDARs (Fig. 2c, d).

### Inhibitory effect of ABs on LTP in the hippocampal CA1 region and NMDAR-mediated calcium influx

The synaptic plasticity of neurons is closely related to learning and memory. We next tested whether tetanus-induced LTP was affected at Schaffer collateral–CA1 synapses in hippocampal slices. In this experiment, CSF from patients or immunized mice was applied for 10 min before theta burst stimulation was applied. Neither of the CSF types affected baseline transmission. The magnitude of LTP at 30 min post-tetanus was significantly smaller with ABs from patients’ CSF (104.0 ± 11.1%, *n* = 5, *P* < 0.005) and GluN1_356–385_ ABs (110.7 ± 8.1%, *n* = 5, *P* < 0.01) than in the control group (151.7 ± 6.2%, *n* = 5) (Fig. 2f). Consistent with these electrophysiological observations, calcium imaging experiments using patients’ ABs or GluN1_356–385_ ABs revealed a marked reduction in NMDAR-induced calcium influx, affecting the total amount of calcium influx (patients’ ABs, *n* = 5, *P* < 0.05; GluN1_356–385_ ABs, *n* = 5, *P* < 0.05) (Fig. 2g).

### Localization of the GluN1 356-385 ABs and their effects in vivo

**Fig. 3:**
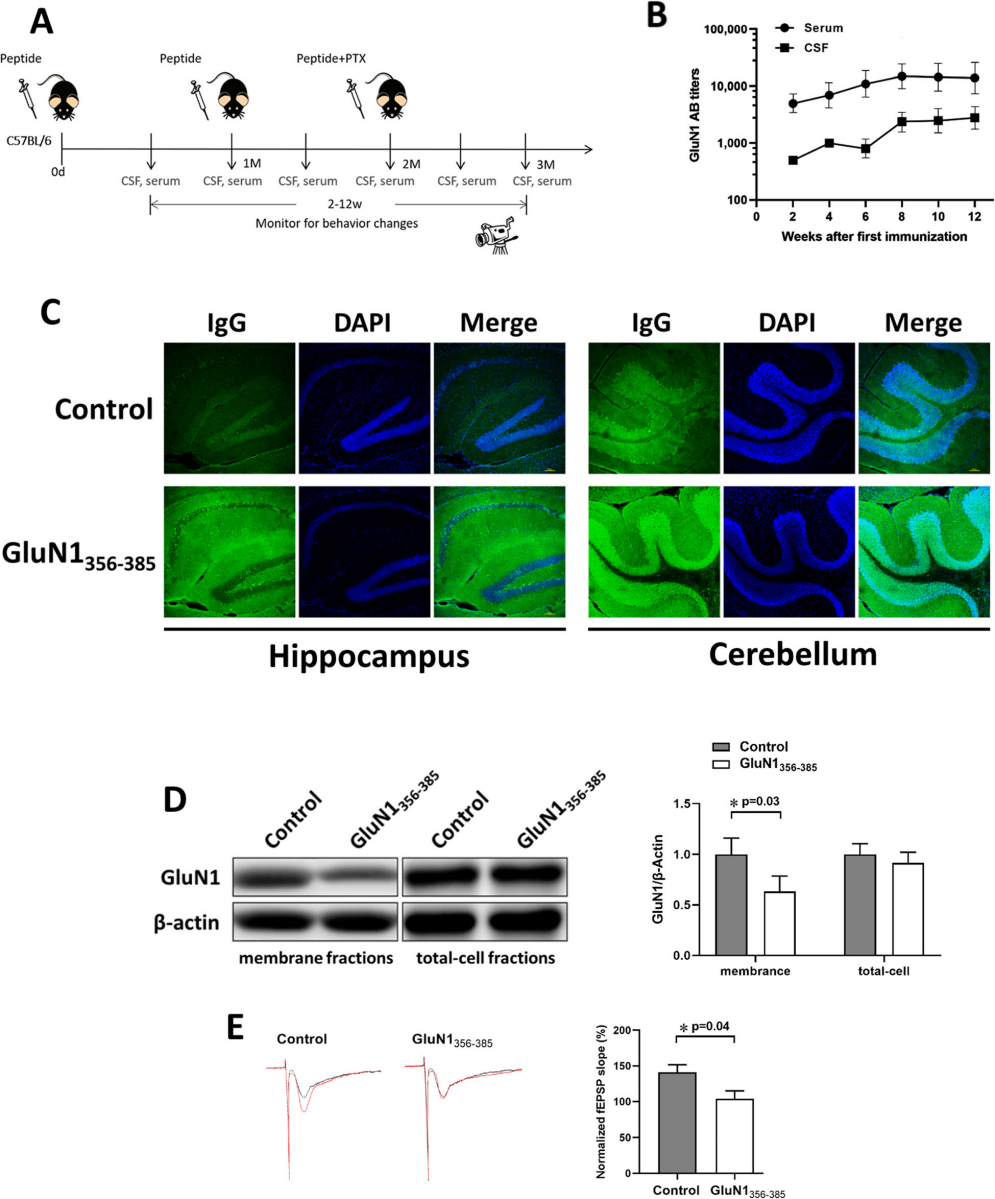
Localization of GluN1_356-385_ ABs and their effects in vivo. **a** The experimental design for active immunization of mice at different time points. **b** CSF and serum AB titers of mice during 12 weeks of immunization (*n* = 3). **c** Immunostaining of mice IgG in brain slices of mice immunized with GluN1_356–385_ peptides. **d** Western blot analysis of GluN1 on membrane fraction and total cell lysates from the hippocampi of immunized mice (*n* = 5). **e** Normalized slope of fEPSP in a hippocampal slice of immunized mice (right). Example traces of fEPSPs recorded before (black) and after (red) TBS (left). *P < 0.05

A functional antibody suggests that it may induce related pathological changes in mice. To verify this, we designed a 12-week experiment to explore whether peptide immunization can induce symptoms in mice. Considering the AB-mediated internalization of NMDARs is a reversible process [7], when the ABs disappear, NMDAR function may be restored. To induce high concentrations of autoantibodies targeting NMDARs, we repeatedly immunized mice with GluN1_356–385_ peptide, with three immunizations at 4-week intervals (Fig. 3a). This procedure maintained the titer of autoantibodies within the CSF and serum of mice (Fig. 3b).

Twelve weeks after immunization, the brains of GluN1_356–385_ peptide-immunized mice were used for immunofluorescence experiments staining for IgG. IgG were detected and mainly distributed in the hippocampus and cerebellum (Fig. 3c). We collected the hippocampal tissues of the immunized mice and extracted membrane proteins to detect the GluN1 expression. Western blotting showed that the membrane amount of GluN1 was significantly reduced after immunization with GluN1_356–385_, indicating a decrease of synaptic NMDARs on the cell surface (Fig. 3d). We further determined whether induced LTP was affected after GluN1_356–385_ immunization. The magnitude of LTP in mice immunized with GluN1_356–385_ (112.0 ± 13.2%, *n* = 5) was significantly reduced compared with the control group (143.1 ± 12.3%, *n* = 5, *P* = 0.04). (Fig. 3e).

### Behavioral changes of mice after immunization with GluN1_356–385_ peptide

**Fig. 4:**
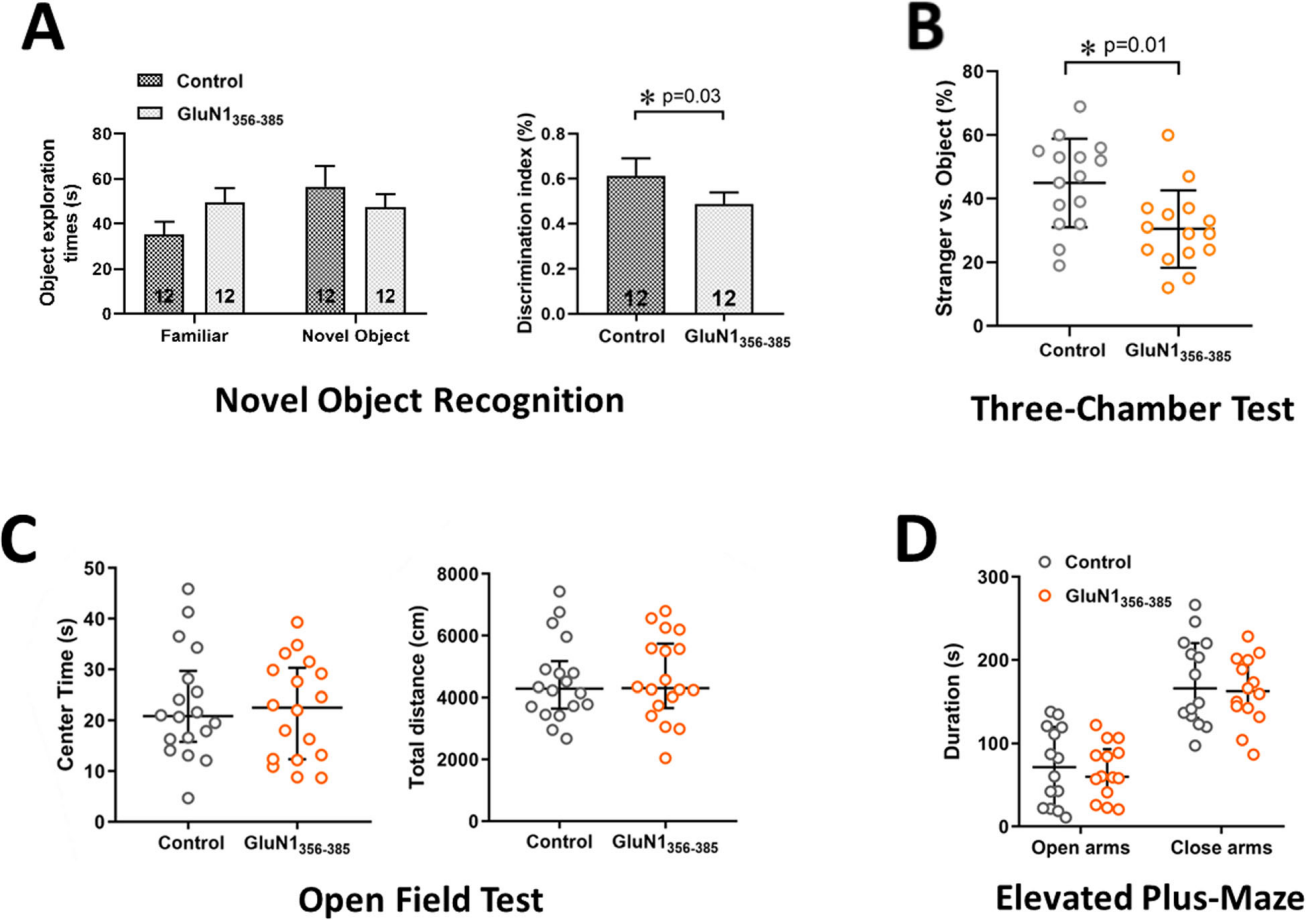
Behavioral changes of mice 12 weeks after immunization with GluN1_356–385_ peptide. **a** Exploration time and Discrimination Index from the NORT of mice 12 weeks after immunization (*n* = 12). **b** 3 chamber test of mice 12 weeks after immunization (*n* = 15). **c** Mice were assessed in the open field for time spent in the center area (left) and for the total distance during a 30-min trial (*n* = 18). **d** Mice were assessed in the elevated plus-maze for time spent in the open arms (left) and close arms (right) during a 5 min trial (*n* = 14)

Symptoms of memory deficits and schizophrenia-like changes are the main presentations that occur in patients with anti-NMDAR encephalitis [4]. To verify the effects of ABs on neurological function and behavioral phenotypes in mice, we performed a series of experiments investigating memory, anhedonia, depressive-like behavior, anxiety, aggression, and locomotor activity in immunized mice. Twelve weeks after immunization, we observed decreased exploration time of the novel object and a lower discrimination index in the GluN1_356–385_ group in the novel object recognition test (NORT) (*n* = 12, *P* = 0.03) (Fig. 4a), in which the immunized mice spent more time exploring the familiar object. The three-chamber experiment revealed that GluN1_356–385_ peptide-immunized mice made fewer visits to strangers than CFA-immunized control mice (*n* = 15, *P* = 0.01) (Fig. 4b). In contrast, there were no differences between the mice in tests of anxiety or depressive behavior tests (open field test and elevated plus-maze) (Fig. 4c, d).

## Discussion

Anti-NMDAR encephalitis is a common form of autoimmune encephalitis, predominantly affecting young adults [17]. Active immunization animal models mimic the process of AB production in autoimmune diseases and have played an important role in the study of neurological diseases [18, 19]. Previous investigations have revealed that peptide fragments can induce autoantibodies to glutamate receptors (AMPA GluN3B), decrease epileptic thresholds, and cause behavioral changes in mice [20]. In the present study, we demonstrated that active immunization with the GluN1_356–385_ peptide targeting the ATD of GluN1 is sufficient to induce high titers of pathogenic anti-GluN1 autoantibodies. This immunization also reproduced many typical anti-NMDAR encephalitis symptoms in mice.

We first determined whether the immunization with GluN1 peptides could induce the production of ABs against GluN1 in the CSF. The ABs from GluN1_356–385_ peptide-immunized mice specifically bound to GluN1-transfected HEK293 cells. In addition, a site mutation at N368Q in the ATD of GluN1 prevented the binding of GluN1_356–385_ ABs to GluN1, suggesting that GluN1_356–385_ ABs share similar epitopes with patient ABs.

Current data support the idea that autoantibodies targeting NMDARs are responsible for disease pathogenesis [21, 22] and CSF titers of NMDAR ABs correlate with clinical relapses of patients [23]. Twelve weeks after immunization, the mice in our study exhibited memory loss, which is consistent with one of the key symptoms of patients with anti-NMDAR encephalitis [1]. Further investigation revealed that treatment with GluN1_356–385_ ABs significantly reduced GluN1 density in the membrane of primary hippocampal neurons and impaired NMDAR function. NMDARs are essential for establishing synaptic plasticity and memory formation. When the hippocampus is targeted by GluN1 ABs, LTP formation at Schaffer collateral–CA1 synapses is severely disrupted [16]. Our electrophysiological results revealed that GluN1 ABs from immunized mice have similar effects. The LTP recordings from CA1 synapses of GluN1_356–385_ AB-treated brain slices showed severe impairment of hippocampal synaptic plasticity, which may explain the memory deficits observed in our animal model.

Studies have shown that autoantibodies targeting NMDARs can induce receptor internalization, thereby affecting NMDAR-mediated electrophysiological activity and transmitter metabolism [24, 25].

At present, the triggering factors for the autoimmune response to NMDARs are still unclear. Previous studies using holoprotein immunogens of tetrameric *Xenopus laevis* GluN1/GluN2B receptors or rat GluN1/GluN2A receptors to produce NMDAR ABs produced fulminant encephalitis [19]. Active immunization of ApoE^−/−^ mice against peptide fragments of GluN1 led to high circulating levels of NMDAR ABs and was able to induce psychosis-like symptoms upon MK-801 challenge [21, 26]. Intranasal infection with herpes simplex virus has also been shown to induce circulating NMDAR ABs [27], which may explain the pathogenesis of secondary anti-NMDAR encephalitis in patients with herpes simplex virus encephalitis. In our study, a peptide from the GluN1 subunit was used as an immunogen. It was sufficient to induce high titers of pathogenic anti-GluN1 autoantibodies. Active immunization also reproduced memory deficits, a typical anti-NMDAR encephalitis symptom in mice. It may be a convenient alternate endogenous model of anti-NMDAR encephalitis that may be useful for further research into the pathogenesis of this disease and aid in the development of potential new therapies.

Our study has several limitations. Although immunized with GluN1_356–385_ peptide decreased NMDAR density in the hippocampus and impaired LTP, the mice did not exhibit mood disorders or spontaneous seizures. GluN1_356–385_ ABs showed binding of the molecular layer of the cerebellum, which is not seen in ABs from patients’ CSF, Additionally, the roles of B cells and T cells in disease induction is still unclear, which should be elucidated with further studies.

## Conclusions

We established a novel anti-NMDAR encephalitis model using active immunization with the peptide GluN1_356–385_, which targets the ATD of GluN1. ABs from GluN1_356–385_ peptide-immunized mice had a similar pathogenic effect to ABs from patients. Compared with the passive AB transfer model, this active immune model better simulates the immunological characteristics of anti-NMDAR encephalitis. We will explore the pathological mechanisms of anti-NMDAR encephalitis using this active immune model in future studies.

## Supplementary Information

**Additional file 1.** Supplementary Methods.

## Data Availability

The datasets analyzed in this study are available from the corresponding authors on reasonable request.

## Abbreviations

NMDAR: N-methyl-D-aspartate receptor
CNS: Central nervous system
CSF: Cerebrospinal fluid
AB: Antibody
ATD: Amino-terminal domain
PBS: Phosphate-buffered saline
HEK293: Human embryonic kidney 293
SDS: Sodium dodecyl sulfate
