# Supplementary material for: Anti-NMDAR encephalitis induced in mice by active immunization with a peptide from the amino-terminal domain of the GluN1 subunit
Source: J Neuroinflammation. 2021 Feb 21;18:53. doi: 10.1186/s12974-021-02107-0 (PMC7897387; doi:10.1186/s12974-021-02107-0)
Supplement: Supplementary file 1 — Additional file 1. Supplementary Methods. [file 12974_2021_2107_MOESM1_ESM.docx]

**Behavioral assessments**

**Novel object recognition test:** The test apparatus consisted of an open dark box made of plexiglass (40 × 40 cm), and the objects were made of plastic and had different shapes. Before the test, the mice were allowed to explore the apparatus for 5 min on 3 consecutive days. On the day of the test, two 5-min tests were performed. First, two identical objects were placed in opposite corners of the box. A mouse was placed in the middle of the box and was left to explore these two identical objects. After a 4 h retention interval, one of the objects was replaced by a new object, and the mouse was allowed to continue exploring for 5 minutes. The time spent exploring each object was recorded. Discrimination Index (DI) was calculated in the following manner:

DI=$\frac{\mathrm{time} \mathrm{spent} \mathrm{exploring} \mathrm{the} \mathrm{novel} object-time \mathrm{spent} \mathrm{exploring} \mathrm{the} \mathrm{familiar} \mathrm{object}}{time spent exploring the novel object+time spent exploring the familiar object}$

**Three-chamber test:** The opaque plastic apparatus consisted of two side chambers of equal size (30 × 30 cm), located on opposite sides of a central chamber and delimited by removable dividers. After 10 min of acclimation to the three-chambered arena, an empty cage and a cage containing an unfamiliar mouse (of the same sex as the mouse being tested) were placed in the opposite side chambers. The locations of the cages were systematically alternated between animals. The time spent in each chamber was recorded over a 10-min period.

**Open-field test:** Each mouse was placed in a 40 × 40 cm square plastic room for 15 min. The total distance traveled and the time spent in the center of the field (15 × 15 cm) were recorded.

**Elevated plus-maze test:** The black plastic apparatus consisted of two closed (10 × 45 × 40 cm) and two open (10 × 45 cm) arms that extended from the same central platform (10 × 10 cm). Each mouse was placed in the center of the maze, facing one of the closed arms, and was kept in the maze for 5 min. The time spent in the open and closed arms and the number of entries per arm were recorded.
